# The joint association of physical activity and sedentary behavior with health-related quality of life among children and adolescents in Mainland China

**DOI:** 10.3389/fpubh.2022.1003358

**Published:** 2022-10-06

**Authors:** Jun Shi, Xiaorong Wang, Qiong Wu, Zhenzhen Qin, Na Wang, Huifen Qiao, Fei Xu

**Affiliations:** ^1^Department of Emergency, Children's Hospital of Nanjing Medical University, Nanjing, China; ^2^Nanjing Municipal Health Institute for Primary and High School, Nanjing, China; ^3^Nanjing Municipal Center for Disease Control and Prevention, Nanjing, China; ^4^Nanjing Brain Hospital Affiliated to Nanjing Medical University, Nanjing, China; ^5^Department of Epidemiology, School of Public Health, Nanjing Medical University, Nanjing, China

**Keywords:** physical activity, sedentary behavior, health-related quality of life, children, adolescent, China

## Abstract

**Objectives:**

The objective of this study was to investigate the joint association of physical activity (PA) and sedentary behavior (SB) with health-related quality of life (HRQoL) among children in Mainland China.

**Methods:**

Children were randomly recruited from primary, junior, and senior high schools (graders 4–12) in Nanjing municipality of China in this cross-sectional survey in 2018. The outcome variable, HRQoL, was assessed using the validated Chinese version of Child Health Utility 9D (CHU9D-CHN) instrument. PA and SB were measured using a validated Physical Activity Scale and Food Frequency Questionnaire for Chinese school-aged children. The associations of PA and SB with HRQoL were assessed using mixed-effects linear regression models and reported as mean difference (MD) and 95% confidence interval (CI).

**Results:**

Totally, 4,388 participants completed the survey (response rate = 97.6%). After adjustment for potential confounders and class-level clustering effects, relative to those with insufficient PA and prolonged SB, students with insufficient PA and shortened SB (MD = 0.054, 95%CI = 0.018, 0.089), or children with sufficient PA and prolonged SB (MD = 0.034, 95%CI = −0.030, 0.098), or students with sufficient PA and shortened SB (MD = 0.083, 95%CI = 0.045, 0.120), respectively, reported increased HRQoL scores.

**Conclusion:**

PA was positively associated with HRQoL, and SB was in negative relation to HRQoL. Furthermore, sufficient PA and shortened SB might exert additive influence on HRQoL among children and adolescents in China. This study has public health implications that PA promotion and SB reduction are encouraged to be considered simultaneously for the purpose to maximally improve HRQoL in population-based behavior intervention campaigns among children and adolescents.

## Introduction

Health-related quality of life (HRQoL) is a single and comprehensive indicator that has been widely used to assess individual's mental and physical health conditions (1–3). Typically, HRQoL refers to self-perception of personal health conditions, including disease symptoms, side effects, functional status within different life aspects, and life satisfaction (1–3). In addition to being used in clinical practice, HRQoL was also used in public health campaigns for effectiveness evaluation of lifestyle and behavior interventions among children and adolescents (4, 5). As two main types of human behaviors, physical activity (PA) and sedentary behavior (SB) were usually involved in these lifestyle and behavior intervention programs targeting on children and adolescents (4, 5). Thus, for the development of population-based HRQoL promotion campaigns through PA and SB intervention among children and adolescents, it is of importance to better understand the association of PA and SB with HRQoL among general children and adolescent population.

With regard to the relationship between PA, SB, and HRQoL among children and adolescents, it has been examined that PA was in positive and SB (predicted by screen time) in negative association with HRQoL, separately, in Western societies (6–10) and China (11). However, there were very few studies to examine the potential joint association between PA, SB, and HRQoL among children and adolescents. Therefore, from the perspective of public health, it is of particular significance to investigate the joint association of PA and SB with HRQoL for the purpose to maximize effectiveness of HRQoL promotion campaigns *via* population-based integrative PA and SB interventions among children and adolescents. To fill this gap, we conducted a school-based study with the main purpose to examine the joint association between PA, SB, and HRQoL among children and adolescents in Nanjing Municipality of Mainland China.

## Methods

### Participants and selection approach

Data analyzed in this study were derived from a cross-sectional survey, The Built Environment and Chronic Health Conditions: Children (BEACH-Children) Study, which was conducted within primary and high school students between May and June of 2018 in Nanjing municipality, a typical mega-city in eastern China. The BEACH-Children Study aimed to explore (1) the relationship between built environment attributes and PA/obesity, (2) the association of lifestyle and behaviors with HRQoL, and (3) the link between health literacy and HRQoL among school-aged children and adolescents in China.

The study city, Nanjing municipality, had 12 administrative districts with more than eight million registered people in the year of 2018 (12). Meanwhile, there were about 667,300 primary and high school students enrolled in the 2017–2018 academic year, and, on average, 40 students within each class (13). Based on the educational system of China, children and adolescents will attend a primary (grades 1–6), junior high (grades 7–9), or senior high school (grades 10–12), while children aged 6 years are eligible to attend grade one. In BEACH-Children study, the participants were graders 4–12 who were randomly selected from all the 12 districts of Nanjing municipality.

These participants were eligible to take part in this study, if they were (1) enrolled in compulsory but not special education schools in all 12 districts of Nanjing municipality, and (2) from grades 4–6 in primary schools, grades 7–9 from junior high schools, or grades 10–12 from senior high schools. However, those students were excluded from the study, if they were (1) with physical disability or/and (2) with severe chronic diseases (e.g., cancers). The estimation of sample size and multi-stage approaches of participants' selection were described in detail elsewhere (14). In brief, the final sample size was determined as about 3,900 to warrant a sufficient statistical power, and 108 classes from 36 schools in the whole city were randomly selected.

The written informed consent was obtained from both participating schools and parents/guardians of eligible participants. The BEACH-Children Study was reviewed and approved by The Academic and Ethics Committee of Nanjing Municipal Center for Disease Control and Prevention, China. The data were de-identified prior to analysis. The methods performed in this study were in accordance with relevant guidelines/regulations recommended by the Declaration of Helsinki.

### Data collection

A standard questionnaire was used to gather data on participants' demographic information and social characteristics as well as parental educational level. Specific sub-scales were applied to collect data on HRQoL, PA, SB, sleep time, fast-food consumption, and sugar-sweetened beverage intake. Before the field survey, research team would discuss with participating schools one by one to appoint a specific survey day for each school. Then, the questionnaire survey was implemented school by school. On each arranged survey day, participants would stay in their regular classrooms as usual and complete the questionnaire survey by themselves. There were a classroom teacher and a research team member available for participants to secure assistance if in need. After the questionnaire survey, participants were organized inside a quiet room to receive objective measurement of body weight and height. Each reading of body weight and height was recorded twice, and then, the mean value was introduced to analysis.

### Study variables

#### Outcome variable

The outcome variable was HRQoL in this study. Participants' HRQoL was measured using the validated Chinese version of Child Health Utility 9D (CHU9D-CHN) (Cronbach's alpha = 0.79) (11). CHU9D is a specific instrument of HRQoL recently developed for assessing effectiveness of clinical treatment or public health interventions among children and adolescents (15). The scoring algorithm of CHU9D-CHN was also validated for Chinese children and adolescents, with a score range from 0 (worst) to 1 (best) (16). In the analysis, CHU9D score was treated as continuous variable.

#### Explanatory variables

There were two main explanatory variables in this study. The first was physical activity. The last-seven-day PA information was collected using the validated Item-specific Physical Activity Scale for Chinese Children and Adolescents (I-PASCA) (Cronbach's alpha = 0.73) (17). The original data on session duration and its weekly frequency of each PA item were reported with I-PASCA by participants. Then, the total time for each PA item in the last 7 days was computed by multiplying session duration and frequency. Next, according to metabolic equivalent (MET) value, each PA item was assigned to a category of intensity: light (MET: <3), moderate (MET: 3–6), or vigorous (MET: ≥6) (18). Finally, the total moderate-to-vigorous PA (MVPA) time (moderate PA time plus doubled vigorous PA time) in the last week was determined and subsequently converted to daily MVPA time. Thus, participants were classified into subgroup of “sufficient PA” if they achieved 60+ min/day MVPA or “insufficient PA” if their daily MVPA time was <60 min based on PA recommendation for Chinese children and adolescents (19).

The second explanatory variable was sedentary behavior. In this study, SB was indicated with screen viewing time in the past week. Moreover, its information was collected with I-PASCA too (17). The session and frequency of screen viewing in the last week were recorded by students. Consequently, the weekly total time of screen viewing was calculated, and the daily screen viewing time was obtained. Then, participants were categorized as “with shortened SB if they viewed screen < 2 h/day” or “with prolonged SB if their screen viewing time ≥2 h/day” according to the specific guidelines for Chinese children and adolescents (20).

Furthermore, for examining the joint association of PA and SB with HRQoL in the study, participants were categorized into one of the four subgroups in the analysis: insufficient PA and prolonged SB (the highest risk group, the reference), insufficient PA and shortened SB, sufficient PA and prolonged SB, or sufficient PA and shortened SB (the lowest risk group).

#### Covariates

Participants' socio-demographic characteristics and some classical covariates were controlled for in the multivariable analysis. Age was treated as a continuous variable, while others were considered as categorical measures. Gender was categorized as boys or girls; residential location was grouped as rural, suburban, or urban area; parental educational attainment was recorded as ≤9 10–12, or 13+years; and school type was classified as primary, junior high, or senior high school.

Sleep time was also measured using the instrument of I-PASCA (17). Each participant was asked to record the total sleep time (hours) in a typical day (including nap time). Then, sleep time was classified as “insufficient” or “sufficient” for analysis according to the age-specific recommendation issued by the National State Council of China (20).

Consumption of fast-food and sugar-sweetened beverage was assessed with a validated food frequency questionnaire (FFQ) (21). This FFQ was specifically developed for measuring dietary intake for children and adolescents in China (21). Data on the intake frequency of fast-food and sugar-sweetened beverage over the previous 7 days were gathered. Due to the very low intake frequency (0.61 serves/week for fast-food consumption and 1.43 serves/week for sugar-sweetened beverage consumption), participants were categorized into either “consuming fast-food or sugar-sweetened beverage (Yes)” or “not consuming fast-food or sugar-sweetened beverage (No)” in the analysis.

Body weight status was assessed based on participants' body mass index (BMI), which was calculated based on objectively measured body weight and height. Then, participants were classified into one of the three subgroups: “underweight/normal,” “overweight,” or “obesity” for analysis based on the age- and gender-specific recommendation for Chinese children and adolescents (22).

#### Data analysis

Differences in selected participant characteristics (percentage) between school types were compared using chi-square test, while differences in HRQoL utility scores [means and standard deviation (SD)] between participant characteristics were analyzed with Student's *t*-test. Two mixed-effects linear regression models were introduced to examine the associations between PA, SB, and HRQoL, with effect estimates reported as mean difference (MD) and 95% confidence interval (CI). Model 1 was a univariable analysis with PA or SB as the only independent variable. Model 2 was a multivariate analysis with PA and SB as independent variable and further adjustment for age, gender, school type, residence, parental educational attainment, body weight status, sleep time, sugar-sweetened beverage, and fast-food consumption. In both analysis models, class-level potential clustering effects were controlled for as a random effect. The significant *p*-value was set as <0.05. Data were managed using EpiData 3.1 (The EpiData Association 2008, Odense, Denmark) and analyzed with SPSS version 20.0 for Windows (SPSS Inc., Chicago, IL, USA).

## Results

### Selected characteristics of participants

Among the 4,498 eligible children and adolescents, 4,388 successfully completed the survey with a response rate of 97.6%. No significant differences were identified between respondents and non-respondents in terms of gender, grade, and residence area. Table 1 presents selected personal characteristics of participants by school type. The mean age (SD) of these participants was 13.9 (2.5) years. The proportion of boys was 50.2%. There were 36.5, 33.2, and 30.3% of children and adolescents from primary, junior high, and senior high schools, respectively.

**Table 1:** Selected characteristics of participants by school type in this study.

**Variables**	**Overall**	**School type**			***p*-value^a^**
	**(*N* = 4,388)**	**Primary school**	**Junior high school**	**Senior high school**	
		**(*N* = 1,602)**	**(*N* = 1,455)**	**(*N* = 1,331)**	
Age					
Mean (SD^b^)	13.9 (2.5)	11.2 (0.9)	14.1 (0.9)	16.9 (0.9)	
Gender (%)					
Boys	50.2	53.9	50.9	45.1	< 0.001
Girls	49.8	46.1	49.1	54.9	
Residence (%)					
Rural	46.3	44.5	46.5	48.1	0.19
Suburban	22.7	24.5	22.2	21.1	
Urban	31.0	31.0	31.3	30.8	
Physical activity (%)^c^					
Insufficient	78.8	70.7	79.5	87.9	< 0.001
Sufficient	21.2	29.3	20.5	12.1	
Sedentary behavior (%)^d^					
≥2 h/day	3.7	3.2	4.7	3.3	0.06
< 2 h/day	96.3	96.8	95.3	96.7	
Body weight status (%)^e^					
Non-overweight	72.3	67.8	73.4	76.6	< 0.001
Overweight	16.7	17.8	16.9	15.2	
Obesity	11.0	14.4	9.7	8.2	
Sleep time (%)^f^					
Insufficient	80.0	77.0	81.0	82.5	< 0.001
Sufficient	20.0	23.0	19.0	17.5	
Sugar-sweetened beverage consumption^g^					
Yes	32.5	29.0	38.1	30.4	< 0.001
No	67.5	71.0	61.9	69.6	
Fast food consumption^g^					
Yes	32.4	37.1	29.1	30.2	< 0.001
No	67.6	62.9	70.9	69.8	

a Chi-square test.

b SD: standard deviation.

c Sufficient physical activity refers to at least 60 minutes/day moderate-vigorous intensity physical activity; while insufficient physical activity means less than 60 minutes/day moderate-vigorous intensity physical activity.

d sedentary behavior: indicated with daily screen time.

e Body weight status was categorized based on age- and gender-specific recommendations for Chinese adolescents.

f Sleeping time: sufficient sleeping time was defined as 10 h/day for children aged 7–13, 9 h/day for children aged 13–16, and 8 h/day for those aged 16–19, based on guidelines for promotion of children and adolescents' physical activity and fitness by The State Council of China.

g Fast-food and sugar-sweetened beverage consumption was classified as “no” or “yes” based on the separate consumption frequency.

### HRQoL scores among participants by socio-demographic characteristics

The mean value (SD) of HRQoL score was 0.78 (0.17) among overall participants. Table 2 displays HRQoL scores among participants by selected characteristics. Boys reported a significantly higher HRQoL score (0.79 ± 0.17) than that recorded by girls (0.78 ± 0.18). The HRQoL score was identified the highest (0.84 ± 0.16) among primary school students but the lowest (0.73 ± 0.18) within senior high school students. Moreover, participants tended to report higher HRQoL score when their parents obtained more educational attainment. However, there was no significant difference in HRQoL scores between participants by residence area.

**Table 2:** HRQoL scores based on CHU9D among participants by selected characteristics in this study.

	**HRQoL Scores**			***p*-value^a^**
	***n***	**Mean**	**SD**	
Overall	4,388	0.78	0.17	N/A
Gender (%)				
Boys	2,204	0.79	0.18	0.017
Girls	2,184	0.78	0.17	
School type				
Primary	1,602	0.84	0.16	< 0.001
Junior high	1,455	0.78	0.16	
Senior high	1,331	0.73	0.18	
Residence (%)				
Rural	2,030	0.79	0.17	0.411
Suburban	997	0.78	0.18	
Urban	1,361	0.78	0.18	
Parental eductaional attainment				
< 9 years	1,264	0.76	0.17	< 0.001
10-13 years	1,415	0.78	0.16	
≥13 years	1,709	0.80	0.18	

a *P*-values for parametric tests between socio-demographic characteristics.

### Separate and joint associations of PA, SB with HRQoL

Table 3 shows the individual and joint associations of PA and SB with HRQoL among participants. Compared to their counterparts with insufficient PA, participants with sufficient PA recorded a significantly higher HRQoL score (MD = 0.037; 95%CI = 0.009, 0.066). Similarly, those children and adolescents with shortened SB reported a significantly higher HRQoL score relative to the participants with prolonged SB (MD = 0.052; 95%CI = 0.013, 0.092). Furthermore, compared to those who were with insufficient PA and prolonged SB, participants either with insufficient PA and shortened SB (MD = 0.058; 95%CI = 0.017, 0.099), sufficient PA and prolonged SB (MD = 0.045; 95%CI = −0.023, 0.114; non-significant), or sufficient PA and shortened SB (MD = 0.094; 95%CI = 0.052, 0.137) perceived higher HRQoL scores, separately. After adjustment for potential confounders, both individual and joint associations of PA and SB with HRQoL attenuated but the association directions remained the same.

**Table 3:** Joint association of physical activity and screen time with HRQoL score among overall participants in this study.

**Exposure variables**		**Participants (*N* = 4,388)**	**Mean (SD)**	**Univariable model** ^*^		**Multivariable model** ^**†**^	
**Physical activity^#^**	**Sedentary behavior**	**% (*n*)**		**Mean difference**	**95%CI**	**Mean difference**	**95%CI**
Insufficient		78.8% (3,459)	0.77	ref.		ref.	
Sufficient		21.2% (929)	0.82	0.037 (0.014)	0.009, 0.066	0.029 (0.011)	0.007, 0.050
	≥2h/day	3.7% (163)	0.73	ref.		ref.	
	< 2h/day	96.3% (4,225)	0.78	0.052 (0.020)	0.013, 0.092	0.050 (0.017)	0.017, 0.084
Insufficient	≥ 2h/day	2.8% (125)	0.72	ref.		ref.	
Insufficient	< 2h/day	76.0% (3,334)	0.77	0.058 (0.021)	0.017, 0.099	0.054 (0.018)	0.018, 0.089
Sufficient	≥2h/day	0.9% (38)	0.76	0.045 (0.035)	−0.023,0.114	0.034 (0.033)	−0.030, 0.098
Sufficient	< 2h/day	20.3% (891)	0.83	0.094 (0.022)	0.052, 0.137	0.083 (0.019)	0.045, 0.120

* Model 1: univariate mixed-effects linear regression model with school class as the random effect.

† Model 2: multivariate mixed-effects linear regression analysis with adjustment for age, gender, school type, residence, parental educational attainment, body weight status, sleep time, sugar-sweetened beverage and fast food consumption, and class-level clustering effects.

# Sufficient physical activity refers to at least 60 min/day moderate-vigorous intensity physical activity; while insufficient physical activity means less than 60 min/day moderate-vigorous intensity physical activity.

## Discussion

In this population-based study, the main aim was to investigate the joint association of PA and SB with HRQoL among children and adolescents in China. It was found that PA was positively associated with HRQoL score, while SB was in negative association with HRQoL score among children and adolescents in China. Furthermore, PA and SB were jointly associated with HRQoL in that sufficient PA and shortened SB might exert additive effect on HRQoL within the participants compared to either sufficient PA or shortened SB separately.

With regard to individual association of PA or SB with HRQoL, it has been reported that a positive PA-HRQoL association and a negative SB-HRQoL link were identified among children and adolescents in Western communities (6–10) and China (11). The findings in our study confirmed the individual relationship between PA, SB, and HRQoL that was examined among children and adolescents in those previous reports. These consistent individual associations of PA and SB with HRQoL among children and adolescents observed from communities with different social and cultural contexts suggest that the association between either PA or SB with HRQoL is likely to hold among general population of children and adolescents worldwide.

There were some potential explanations behind the associations of PA and SB with HRQoL among children and adolescents. It has been documented that PA could improve children's desirability of involvement in social activities and their social functioning (23), while, on the contrary, SB was in negative association with social participation and activity for children and adolescents (24, 25). Meanwhile, social involvement and activity were positively associated with HRQoL (26). Thus, this might, at least partly, underlie the associations of PA and SB with HRQoL among children and adolescents identified in previous studies and our survey.

Furthermore, it is reasonable to applaud that the more PA and the less SB may be jointly associated with the better self-perceived HRQoL based on these two mechanisms. This was the case observed in our study. Sufficient PA and shortened SB might be associated with much better HRQoL compared to each of them, separately, among children and adolescents, which suggested an additive effect of sufficient PA and shortened SB on HRQoL. Such a finding is of public health significance, as it implies that particular attention shall be paid to PA promotion and SB reduction simultaneously for the purpose to improve HRQoL among children and adolescents through behavior intervention campaigns.

The findings from this study are not only of interest for academic researchers but also of realistic instructive significance for public health practitioners. For researchers, they are welcome to further investigate the potential causal relationship between PA, SB, and HRQoL among children and adolescents with well-designed studies, for example, longitudinal studies, under different social and cultural contexts. For example, it is of help to establish a surveillance system to continuously collect data on PA, SB, and HRQoL based on cohorts of students. In this way, it is possible to examine the potential causal association of PA and SB with HRQoL among students and to evaluate the population-level trajectory of PA, SB, and HRQoL for students. On the contrary, for public health practitioners, they are encouraged to work together with educational staff to implement regular school-based PA promotion and SB reduction campaigns simultaneously for the purpose to improve HRQoL among students. Moreover, such school-based PA promotion and SB reduction campaigns shall be integrated into schools' arrangements as fixed plans.

Some strengths of this study were worthy of being noted. First, a representative sample of general population of children and adolescents was randomly chosen from all areas of a typical mega-city in China. Second, 97.6% of eligible participants completed the study, implying the generalizability of the findings in this study. Third, a specifically validated instrument was applied to assess HRQoL, while its scoring approach was also specifically developed for Chinese children and adolescents. Finally, interesting findings, a potentially additive effect of sufficient PA and shortened SB on HRQoL, were observed.

Several limitations of this study should also be addressed. First, PA and SB were measured with self-reported questionnaire, although they were validated. Second, the nature of cross-sectional survey could not warrant any causality of the associations of PA and SB with HRQoL in the study. Last, due to lack of data, only a few confounders (socio-demographic attributes, anthropometric characteristics, and lifestyle factors) were adjusted for in the analysis. Therefore, the findings observed in this study should be interpreted prudently.

## Conclusion

Physical activity was positively associated with HRQoL, and sedentary behavior was in negative relation to HRQoL. Furthermore, sufficient physical activity and shortened sedentary behavior might exert additive influence on HRQoL among children and adolescents in China. This study has public health significance that physical activity promotion and sedentary behavior reduction are encouraged to be considered simultaneously for the purpose to maximally improve HRQoL in population-based behavior intervention campaigns among children and adolescents.

## Data availability statement

The original contributions presented in the study are included in the article/supplementary material, further inquiries can be directed to the corresponding authors.

## Ethics statement

The studies involving human participants were reviewed and approved by the Ethics Committee of Nanjing Municipal Center for Disease Control and Prevention. Written informed consent to participate in this study was provided by the participants' legal guardian/next of kin.

## Author contributions

HQ and FX conceived and designed the broad study project. QW, ZQ, NW, and FX collected data of the BEACH-Children study. JS, XW, QW, ZQ, NW, HQ, and FX conceived, discussed, designed the work presented in this manuscript, and wrote and critically reviewed the manuscript. ZQ and FX analyzed the data presented in this manuscript. All authors approved the final version for submission and was also responsible for all aspects of the work presented in this manuscript.

## Funding

This work was supported by a key grant from Nanjing Medical Science and Technique Development Foundation (ZKX16052: Recipient: HQ), Nanjing Municipal Science and Technique Development Foundation (201715058: Recipient: ZQ), and Nanjing Medical Science and Technique Development Foundation, China (QRX11038: Recipient: FX). Both Nanjing Medical Science and Technique Development Foundation and Nanjing Municipal Science and Technique Development Foundation had no role in the design and conduct of the study; collection, management, analysis, and interpretation of the data; preparation, review, or approval of the manuscript; and decision to submit the manuscript for publication.

## Conflict of interest

The authors declare that the research was conducted in the absence of any commercial or financial relationships that could be construed as a potential conflict of interest.

## Publisher's note

All claims expressed in this article are solely those of the authors and do not necessarily represent those of their affiliated organizations, or those of the publisher, the editors and the reviewers. Any product that may be evaluated in this article, or claim that may be made by its manufacturer, is not guaranteed or endorsed by the publisher.
